# Chemical Composition of *Litsea pungens* Essential Oil and Its Potential Antioxidant and Antimicrobial Activities

**DOI:** 10.3390/molecules28196835

**Published:** 2023-09-27

**Authors:** Tao Chen, Qingbo Kong, Xuekun Kuang, Jiasi Zhou, Haizhou Wang, Lijun Zhou, Hongyu Yang, Shiling Feng, Chunbang Ding

**Affiliations:** College of Life Science, Sichuan Agricultural University, Ya’an 625014, China; chentao293@163.com (T.C.); kqb666666@163.com (Q.K.); larrymercer11@gmail.com (X.K.); zhoujiasi1999@163.com (J.Z.); wanghaizhou4678@163.com (H.W.); zhoulijun@sicau.edu.cn (L.Z.); yhy4868135@163.com (H.Y.); fengshilin@outlook.com (S.F.)

**Keywords:** *Litsea pungens*, antioxidant, antimicrobial, essential oil

## Abstract

*Litsea pungens* is a plant with medicinal and edible properties, where the fruits are edible and the leaves have medicinal properties. However, there is limited research on the chemical and pharmacological activities of the plant. In this study, essential oils were extracted by steam distillation and their antioxidant and antibacterial activities were further evaluated. Gas chromatography–mass spectrometry (GC–MS) was used to identify the chemical components of *L. pungens* fresh fruit essential oil (FREO) and *L. pungens* fresh flower essential oil (FLEO), rapeseed oil (RO) and commercial *Litsea* oil (CEO). The results showed that 12 chemical components were identified in FREO. Twelve chemical components were identified from FLEO, four chemical components were identified from CEO, and thirteen chemical components were identified from RO. Except for RO, the other three oils were mainly composed of terpenes, among which limonene is the main chemical component. In terms of antioxidant activity, FREO, FLEO, CEO and RO have antioxidant capacity, mainly reflected in the scavenging DPPH free radicals and the iron ion chelating ability, and the antioxidant activity shows a certain dose effect, but the antioxidant activity of FLEO is the weakest among the four oils. Meanwhile, under the stress of hydrogen peroxide, CEO demonstrated a significant antioxidant protective effect on cells. It is worth mentioning that compared with the positive control, the FREO exhibited a better antibacterial rate. When the concentration of essential oil is 20 mg/mL, the bacteriostatic rate can reach 100%. Therefore, it could be a promising candidate among medicinal and edible plants.

## 1. Introduction

Over recent decades, plant natural products have garnered significant attention from researchers and scientists due to their diversity, biological activity, sustainability and ecological friendliness [1]. Essential oils (EOs) are mixtures of aromatic volatile secondary metabolites with natural properties that not only surpass synthetic chemicals in terms of efficiency and safety, but also possess a wide array of medical and biological properties [2]. The current great interest in the study of EOs is for their medical and biological properties, as they are generally considered safe and have the potential to work synergistically with other substances, which are attractive for their use as bioactive molecules [3]. The chemical components of EOs mainly include aromatics, terpenes, terpenes, esters, etc. The content of each component gives the EOs specific biological activities [4]. EOs have been reported to have antioxidant [5], anti-inflammatory [6], antibacterial [7,8], anti-tumor [9] and allelopathic effects [10,11]. The antioxidant capacity of oil is attributed to its ability to reduce oxidation reactions and reduce the amount of free radical generation. Therefore, it can be preliminarily used for antioxidative stress induced damage. At present, Clove essential oil and olive oil have been proved to have strong antibacterial and antioxidant properties [12,13].

*Litsea pungens* is a deciduous shrub or small arbor of the *Litsea* genus and family Lauraceae, it is one of the unique spice plant resources in China. It is mainly distributed in China, India, Indonesia and other countries, with a major concentration in southern China, especially along the Yangtze River [14]. Its fruit, leaves and branches contain essential oils. It has been reported that the EOs extracted from aromatic plants have a variety of biological properties. A plant essential oil is composed of at least ten different chemical components, making it difficult for some microorganisms to simultaneously resist all active components in the essential oil [15]. In addition, food spoilage and cell damage may be due to the excessive production of reactive oxygen species. Synthetic chemical inhibitors are commonly employed to control this oxidative phenomenon, but they have significant negative effects on human health. Natural secondary metabolites, including EOs, have been shown to possess significant biological activities [4]. So far, there are many studies on *L. cubeba* essential oil, mainly focusing on antibacterial, antioxidant and anti-inflammatory aspects [16,17].

As per current knowledge, there is a scarcity of research on the antioxidant and antimicrobial activity exhibited by the essential oil of *L. pungens*. To this end, the essential oil of the flower and fruit of *L. pungens* was extracted by steam distillation and the chemical constituents of the essential oil, commercial *Litsea* oil (CEO) and rapeseed oil (RO) were further analyzed by gas chromatography–mass spectrometry (GC–MS). In addition, the biological activity potential of the essential oil was evaluated for the first time, and its antioxidant activity was evaluated by measuring its free radical scavenging ability and iron ion chelating ability. Meanwhile, the antibacterial activity of essential oil was also evaluated. Therefore, *L. pungens* exhibits potential as a viable candidate in the realms of medicine and food.

## 2. Results and Discussions

### 2.1. The Composition of Essential Oils of L. pungens

**Figure 1:**
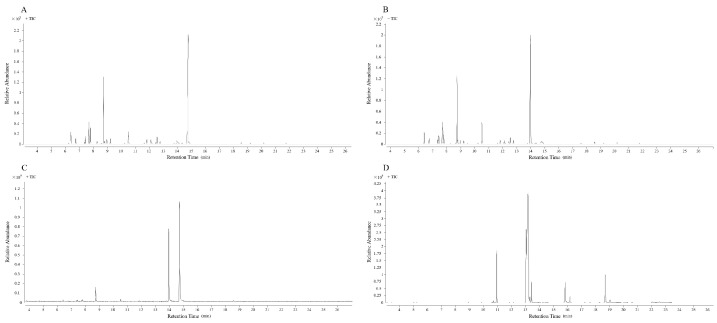
Gas chromatography–mass spectrometry of four oils ((**A**): FREO; (**B**): FLEO; (**C**): CEO; (**D**): RO).

**Figure 2:**
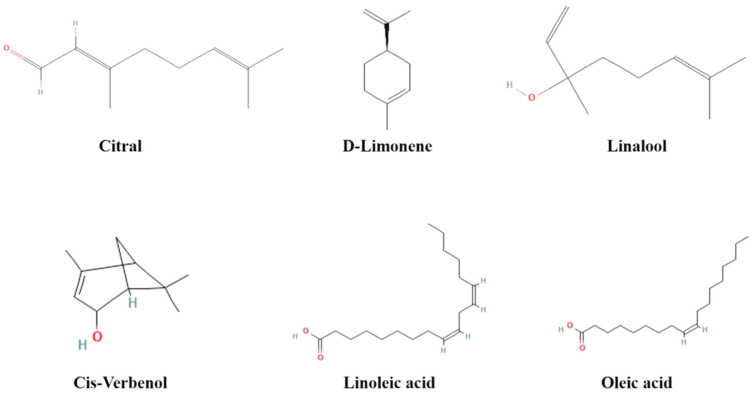
Molecular structure of some major phytochemicals identified in four oils.

**Figure 3:**
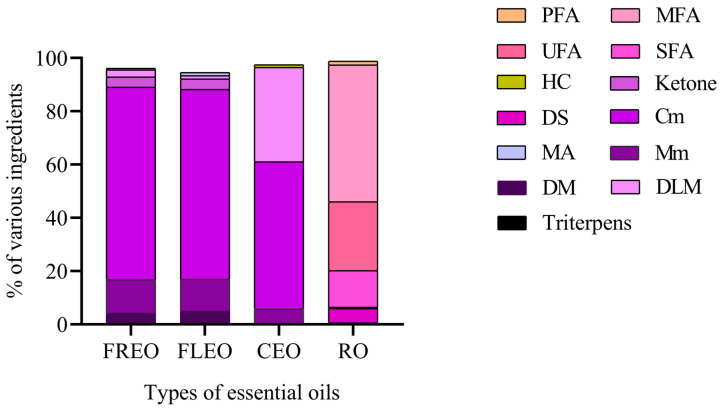
Components of the chemical classes from four oils. (FREO: fresh fruits essential oil; FLEO: fresh flower essential oil; CEO: commercial *Litsea* oil; RO: rapeseed oil; PFA: Polyunsaturated fatty acid; MFA: Monounsaturated fatty acid; UFA: Unsaturated fatty acid; SFA: Saturated fatty acid; Cm: Chain monoterpene; DLM: Double loop monoterpene; HC: Hydrazine compound; DS: Dicyclic sesquiterpene; MA: Monoterpene alcohol; Mm: Monocyclic monoterpene; DM: Dicyclic monoterpene).

The EOs were obtained by steam distillation and the constituents of four oils were identified by GC–MS. The chromatographic graph of the chemical constituent of the EOs is presented in Figure 1 and further details can be found in Table 1, as well as Figure 2 and Figure 3. A total of 12 compounds were detected in the essential oil of fresh fruits (FREO). The main component was chain monoterpene, with citral accounting for 68.23%. Twelve compounds were detected in fresh flower essential oil (FLEO), among which citral accounted for 66.69% of the total essential oil, followed by D-limonene (11.96%). The main component of CEO was citral from chain monoterpenes, accounting for 55.29% of the total essential oil, followed by a Cis-verbenenol content of 35.36%. Finally, 13 compounds were detected in RO, which were mainly monounsaturated fatty acids, followed by unsaturated fatty acids. Among them, oleic acid constituted 45.30% of the monounsaturated fatty acids, while linoleic acid was the most abundant among the unsaturated fatty acids.

Except for RO, the other three oils were mainly composed of terpenes, among which limonene is the main chemical component. Limonene is widely used as a flavor additive in beverages, food, fragrances and cosmetics. Limonene has been found to have beneficial therapeutic effects such as anti-inflammatory, antioxidant and anticancer effects [18]. In previous studies, there were more studies on *L. cubeba*, but there were fewer studies on *L. pungens*. Although both belong to *Litsea*, there are differences in their odor and composition category. According to the analysis of the chemical components of *L. cubeba* essential oil by Thielmann et al., the main components were citral and limonene and it had a broad-spectrum antibacterial effect [19]. Another report found that the content of D-limonene in the *L. cubeba* fruit essential oil was low (0.7~5.3%) and its main components were citral and vanillin [20]. In total, 33 compounds were identified from the essential oil of *L. cubeba* fruit, and the relatively high contents were limonol (44.2%), β-linalool (8.8%), 1,8-cineol (5.4%) and elemelemin (3.9%) [21]. The current results are still different from previous reports, which may be due to different varieties, environmental factors and geographical distribution [17,22].

### 2.2. Antioxidant Ability

**Figure 4:**
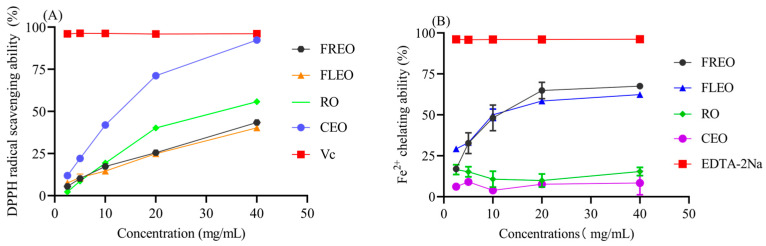
The antioxidant ability of four oils on DPPH free radical scavenging capacity (**A**). The Fe^2+^ chelating ability (**B**).

In this study, the antioxidant activity of four oils was estimated by determining their ability to scavenge DPPH and chelate Fe^2+^ ions. As shown in Figure 4A, *L. pungens* essential oil possessed antioxidant capacity. Among the four oils tested, CEO exhibited the best DPPH radical scavenging ability, followed by RO, FREO and FLEO. The clearance of DPPH increased with higher concentrations of the oils, indicating a clear dose–effect relationship. Wang et al. also found that the essential oil from dry fruits of *L. cubeba* showed a notable antioxidant ability on hydroxyl radical and superoxide [22]. She et al. observed that the antioxidant ability of *L. cubeba* essential oil varied depending on the month of fruit collection [23]. In addition, other studies have shown that limonene has strong antioxidant capacity [24], and citronellal (60.66%) extracted from *E. citriodora* leaves also demonstrated formidable antioxidant capacity [25]. In this study, the CEO exhibited stronger antioxidant activity than the other three oils. This superior activity may be attributed to the high content of citral, Cis-verbenol and D-Limonene in the CEO, with the verbenol content being the highest among the three oils. However, it remains unclear whether the enhanced antioxidant activity results from a synergistic effect of the chemical components or if a single component plays a dominant role. Future studies will aim to elucidate the specific pathways of action and potential in these compounds. Furthermore, the CEO was extracted using RO, while the other two EOs were obtained through distillation extraction methods. The clearance ability of RO was comparable to that of the other Eos, but lower than that of the CEO (Figure 4A). These different extraction methods extract distinct chemical compositions, which may also contribute to the higher antioxidant activity observed in the CEO [26,27]. Hence, components with excellent antioxidant capabilities were extracted from *L. pungens* using RO.

As the antioxidant ability of essential oil was further investigated, however, CEO and RO showed a weak Fe^2+^ chelating ability and two essential oils, nevertheless, exerted a strong chelating ability (Figure 4B). Among those EOs, FREO showed a stronger chelating ability than the other three EOs, indicating some compositions with a great chelating capacity were extracted by the distillation extraction method. Fe^2+^ is a catalytic metal ion. In addition to initiating lipid peroxidation through the Fenton reaction, it can also trigger arthritis and cancer. Fresh essential oils could well chelate Fe^2+^, indicating the essential oils are probably suitable for eating to prevent cancer [28].

### 2.3. The CEO Protected CHO Cells against H_2_O_2_

**Figure 5:**
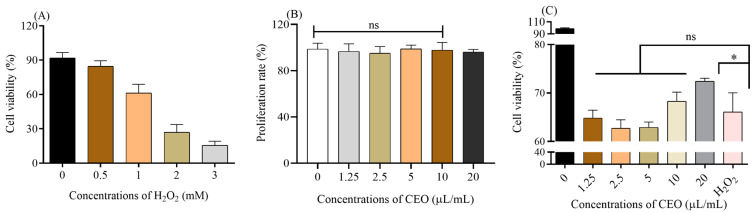
The protective impact of CEO on cellular oxidative damage; (**A**) The H_2_O_2_ injury model; (**B**) The toxicity of CEO on CHO cells after 48 h treatment; (**C**) The cell viability of CHO treated with CEO under oxidative condition. Differences were considered to be significant at *p* < 0.05 (*).

Since CEO exhibited an excellent DPPH free radical scavenging ability, the protection effect of CEO on a cellular level was further investigated. Different concentrations of H_2_O_2_ were added into CHO cells and the cell viability was between 60 and 70% under 1 mM H_2_O_2_, indicating the injury model was successfully established (Figure 5A). Hence, 1 mM H_2_O_2_ was used for the following assays. The cytotoxicity of CEO on CHO were shown in Figure 5B. Within the concentration range in this work (0–20 μL/mL), the CEO has no toxic side effects on the proliferation of CHO cells. Meanwhile, the cell viability of CHO cells was quite different under H_2_O_2_ conditions after pretreatment with CEO for 48 h. The cell viability (1.25–10 μL/mL) was not significantly different from the injury group (*p* > 0.05), but 20 μL/mL CEO could significantly increase the cell survivals compared with the injury group (Figure 5C).

As a messenger molecule, H_2_O_2_ spreads through cells and tissues and immediately triggers cellular effects such as cell shape change, initiation of cell proliferation, and recruitment of immune cells [29]. Meanwhile, H_2_O_2_ was a trigger for reactive oxygen species (ROS). When the body is in an oxidative stress environment, a large amount of ROS will be produced, which will induce the excessive production of free radicals, which may induce lipid peroxidation, cell aging, DNA damage and other hazards [30]. Previous studies have shown that many monoterpenes (camphor, eucalyptol) act as biological anti-mutagenic agents by stimulating DNA repair [31], or as anti-mutagenic agents (i.e., Linalool, myrcen) to prevent DNA damage [32,33]. In this study, the ROS level increased in the injury group compared with that in the control group. When the cells were pretreated with CEO for 48 h, the ROS level in the cell sharply decreased, indicating CEO could stimulate the antioxidant system to clear the increasing ROS. The main chemical components of the CEO are citral (55.29%) and Cis-verbenol (35.36%), both of which belong to monoterpenoids, and they play a protective role in oxidative damage. Therefore, from the above results, CEO has strong antioxidant protection at the cellular level.

### 2.4. Antimicrobial Activity

As can be seen from Table 2, the inhibitory effects of FREO on the two fungi are different. Compared with the blank control and negative control, the bacteriostatic rate was significantly different. With the increase of the concentration of the essential oil, the antibacterial rate gradually increased, showing a dose effect. At the concentration of 20 mg/mL, the two kinds of fungi were completely inhibited by FREO and the inhibition rate reached 100%. Compared with the positive control group, the inhibitory rate of the positive control group was weaker than the maximum concentration (20 mg/mL). In addition, the inhibitory effects of FLEO on the two types of fungi were also different. When the concentration of the essential oil was 10 mg/mL, the inhibitory rate of fusarium wilt reached 42.90%, while that of anthrax was only 13.03%, indicating that the essential oil was more sensitive to fusarium wilt. In addition, when the concentration of the essential oil was 20 mg/mL, the inhibitory rates of the essential oil against the two kinds of fungi were 86.05% and 92.45%, respectively, which had no significant difference compared with the positive control. In summary, the antibacterial activity of FREO was stronger than that of FLEO. According to previous research findings, EOs extracted from eucalyptus leaves have been shown to have significant inhibitory effects against a variety of plant pathogens, including *C. gloeosporioides* [34]. The essential oil extracted from *Eucalyptus grandis* × *E. urophylla* leaves by the supercritical CO_2_ method adopted by Zhou et al. has excellent antifungal effects on a variety of plant pathogenic fungi, including *Fusarium graminearum* and *Fusarium moniliforme* [35]. Liu et al. found that the terpenoids in the essential oil of *Eucalyptus grandis* × *E. urophylla* had inhibitory effects on *Fusarium oxysporum* and *Glorosprium musarum* [36].

Interestingly, at the same concentration and treatment time, the overall antibacterial rate of FREO was stronger than that of FLEO on both fungi. The reason for the difference in the antibacterial rate of the two EOs should be the main chemical component content (citral and D-limonene) and other components. Chang et al. found that limonene and α- and β-pinene had strong inhibitory effects on *C. gloeosporioides* and other fungi [37]. At the same time, An et al. also found that terpene-4-ol and α-terpinol could effectively inhibit mycelial growth and spore germination [38]. Citral, cinene, α- and β-pinene, α-terpinol and other major components were found in the *L. pungens* essential oil of the fresh fruit and the fresh flower, but their contents were different. Therefore, we preliminarily speculate that these main chemical components play an important role in the antifungal activity of the essential oil, but the specific antibacterial mechanism of the essential oil needs to be further studied.

## 3. Materials and Methods

### 3.1. Plant Materials and Reagents

The fruits and flowers of *L. pungens* were collected in March and September 2022 from Hongya County, located in Sichuan Province, China. The commercial *Litsea* oil (CEO) and rapeseed oil (RO) were purchased from Sichuan Hongya County Yaomazi Food Co. Ltd. (Meishan, China).

2,2-Diphenyl-1-picryl-hydrazyl (DPPH) was obtained from Sigma Chemical Co. (St. Louis, MO, USA). Ethanol, dimethyl surtoxide (DMSO), ferrous sulfate and sodium sulfate were bought from the Chengdu Kelong Chemical Factory (Chengdu, China). Reactive Oxygen Species Assay Kit (ROS) was gained from Beyotime Biotechnology (Shanghai, China). Cell counting kit-8 (CCK-8) was bought from Nanjing Jiancheng Bioengineering Institute (Nanjing, China). FBS was purchased from Gibco. Dulbecco’s modified eagle medium (DMEM), PBS, digestive enzyme and Penicillin–Streptomycin solution (10×) were bought from Hyclone (Logan, UT, USA). All chemicals were analytical grade.

### 3.2. Extraction of L. pungens Essential Oil

The *L. pungens* fresh fruit and flower were cut into pieces and extracted by steam distillation for 6 h, respectively. After standing and layering, anhydrous sodium sulfate was added to remove water, then the essential oil was obtained and stored in a brown bottle at 4 °C until analyzed by gas chromatography–mass spectrometry and bioassays.

### 3.3. Gas Chromatograph/Mass Spectrum (GC–MS) Analysis

The chemical compositions of EOs were determined by GC-MS (Agilent Technologies, Palo Alto, CA, USA). First, 1 µL essential oils was resolved in chromatography n-hexane (ratio of 1/10). Gas chromatographic conditions: HP-5MS 5% Phenyl Methyl SiIox (30 mm × 0.25 mm, 0.25 μm) chromatography column, carrier gas was high-purity helium (99.99%), using a temperature program. The temperature was held at 60 °C for 3 min and then raised to 100 °C at 12 °C/min and kept for 5 min. The temperature was raised to 160 °C at 10 °C/min and kept for 5 min. Subsequently, the temperature was raised to 280 °C at 12 °C/min and kept for 5 min. The carrier gas was helium and a 1 μL sample was injected in a non-shunt mode. The injection port temperature was 260 °C and the interface temperature was 220 °C. Mass spectrometry conditions: electron bombardment (EI) ion source. Electron energy was 70 eV and electron multiplier voltage was 1.5 kV. Mass scanning ranged from 40 to 600 (*m*/*z*) fulling scanning.

### 3.4. Identification of Essential Oil Chemical Conponents

The software AMDIS v2.71 (www.amdis.net) (accessed on 5 June 2023) was employed to analyze and identify the essential oils. The chemical structure of each peak mass spectrum was determined by comparing the mass spectrum data retrieved from NIST spectrum library and combining with the retention index. The relative contents of essential oil components were calculated by the normalized area method [39,40].

### 3.5. Antioxidant Assays

#### 3.5.1. DPPH Clearance Ability

Essential oils of *L. pungens* fresh fruit and flower and RO were resolved in ethanol to different concentrations (0~40 mg/mL). The DPPH free radical scavenging ability was determined by previous method with some modifications [41]. Briefly, 100 μL sample solution was mixed with 100 μL DPPH-ethanol solution (0.2 mM) for 30 min in dark. Ascorbic acid (Vc) was used as positive control. Then the absorbance was read at 517 nm. The DPPH free radical scavenging ability was calculated according to the following formula:

DPPH free radical scavenging ability (%) = 1 − A_1_/A_2_

where A_1_ = was the absorbance of sample and DPPH mixed solution and A_2_ = was the absorbance of DPPH and ethanol instead of mixed solution.

#### 3.5.2. Fe^2+^ Chelating Ability

Four oils were dissolved in ethanol and diluted to different concentrations. The chelating ability of Fe^2+^ was determined by previous method [42]. A total of 50 μL of sample solution was added into 100 μL ferrous sulfate solution (0.125 mM) and 50 μL ferrotine solution (1 mM), then the mixture was placed at 37 °C for 30 min in dark. Ethylenediaminetetraacetic acid disodium salt (EDTA-2Na) was used as positive control. Subsequently, the absorbance of each mixture was read at 562 nm. The chelating ability of Fe^2+^ was calculated according to the following formula:

Fe^2+^ chelating capacity (%) = 1 − (A_1_ − A_2_)/A_3_

where A_1_ = was the absorbance of sample, ferrous sulfate and ferrotine mixture solution; A_2_ = was the absorbance of sample, ferrous sulfate and distilled water instead of mixed solution; A_3_ = was the absorbance of ethanol in replaced sample solution, ferrous sulfate and ferrotine mixed solution.

#### 3.5.3. Cell Culture

Chinese hamster ovary cells (CHO) were kindly provided by the Biotechnology Center of Sichuan University (Chengdu, China). CHO cells were stored in DMEM containing 10% FBS at 37 °C in 5% CO_2_ atmosphere. When cells reached 70–80% confluence, CHO cells were digested from the flask and washed with PBS twice before planted onto plate.

#### 3.5.4. Establishment of H_2_O_2_-Induced Model

CHO cells were digested from the bottle and adjusted to 10^4^ cells/mL [43]. Then, 90 μL suspension was added to 96-wells plate for 6 h. Next, 10 μL H_2_O_2_ solution of different concentrations was put into it for another 5 h at 37 °C. Subsequently, 100 μL fresh medium containing 10% CCK-8 was replaced with the solution. The plate was placed in dark for 30 min before reading the absorbance at 450 nm. The cell viability was calculated according to the following formula:

Cell viability (%) = 1 − A_1_/A_2_

where A_1_ = was the absorbance of group treated with sample solution and A_2_ = was the absorbance of group treated with equal volume medium.

#### 3.5.5. Toxicity of Essential Oil on CHO Cells

CEO was dissolved in DMSO and filtered through 0.22 μm filter membrane. Cell suspension was inoculated into 96-wells plate at the density of 10^4^ cells/well. Different concentrations of oil were added into each well after 6 h cultivating. Then the plate was placed at 37 °C for 48 h. Next, 100 μL new medium containing 10% CCK-8 kit was transferred into the plate after removing the initial medium. The absorbance of the plate was read at 450 nm after incubation for 30 min. The cell viability was calculated to evaluate the toxicity of essential oil on CHO cells.

Cell viability (%) = A_1_/A_2_ × 100

where A_1_ = was the absorbance of the cell well with cells, CCK-8 solution and sample solution; and A_2_ = was the absorbance of a well with cells, CCK-8 solution and PBS replaced with sample solution.

#### 3.5.6. Protection of Essential Oil on CHO Cells under H_2_O_2_ Stress

CHO cells were inoculated onto plate for 6 h, then different concentrations of essential oil were transferred to plate making the terminal concentration of DMSO less than 0.5%. After incubation for 48 h at 37 °C, the solution was removed and added to H_2_O_2_ for 5 h. Then 100 μL fresh medium containing 10% CCK-8 replaced H_2_O_2_ solution. The absorbance of each well was read at 450 nm to determine cell viability.

Cell viability (%) = A_1_/A_2_ × 100

where A_1_ was the absorbance of the cell well with cells, CCK-8 solution and sample solution; and A_2_ = was the absorbance of a well with cells, CCK-8 solution and PBS replaced with sample solution.

### 3.6. Antifungal Assay

The antifungal activity of essential oil was evaluated by growth rate method [35]. Essential oil resolved by DMSO was added to PDA medium to prepare essential oil PDA culture plate with final concentration of 2.5, 5, 10 and 20 mg/mL. Distilled water group was used as blank control (CK), DMSO solution group as negative control (CK1) and 20 mg/mL carbendazim solution as positive control (CK2). The strains were punched into 6 mm fungus cake. The inoculation shovel transferred the prepared piece on the medium as the mycelial side was down. Then the medium was incubated at 25 °C. The mycelial growth was observed every day and the diameter of each culture plate was measured with Vernier caliper (the average value was measured by cm by cross method three times) until the culture plate of the blank control was full of mycelium. The inhibitory effect was evaluated uniformly at 96 h after inoculation (the day before the blank control plates were fully covered with mycelium). The inhibition rate was calculated as follows:

Inhibition rate (%) = (A_0_ − A_1_)/(A_0_ − 6)

where A_0_ was the diameter of blank control and A_1_ was the diameter of test group.

### 3.7. Statistical Analysis

Experiments were carried out in triplicates and analyzed statistically by ANOVA (GraphPad Prism 6) (GraphPad Software, Inc., La Jolla, CA, USA). All results were expressed as mean ± standard error of the mean (SEM), *n* = 3. 

## 4. Conclusions

In this study, except RO, the main chemical components of the other three oils are terpenes, with citral being the highest in content. In terms of antioxidant activity, the essential oils from fresh flowers and fruits of *L. pungens*, CEO and RO exhibit similar antioxidant capacities. This is mainly observed in their ability to scavenge DPPH free radicals and chelate iron ions. The antioxidant activity also demonstrates a certain dose effect. However, the FLEO shows the weakest antioxidant activity among the four oils. Simultaneously, when exposed to hydrogen peroxide stress, CEO exhibits effective antioxidant protection on cells. Additionally, in terms of antibacterial activity, the activity of FREO is stronger than that of FLEO. It is worth mentioning that the antibacterial rate of FREO is superior to that of the positive control. When the concentration of essential oil is 20 mg/mL, the bacteriostatic rate can reach 100%, but the specific bacteriostatic mechanism will be developed in the next step. The preliminary confirmation of the antioxidative and bacteriostatic effects of the essential oil has been established. Additionally, the results of this study have laid a theoretical foundation for the further development and utilization of *L. pungens*. It is believed that *L. pungens* has extensive potential application in the fields of food and medicine.

## Figures and Tables

**Table 1:** The chemical composition of EOs of *L. pungens* and CEO and RO.

Type of Essential Oil	No.	RT	RI	Relative Contents (%)	Identified Compounds	Type of Compounds
FREO	1	6.399	922	2.00	α-Pinene	DM
	2	6.749	943	0.90	Camphene	DM
	3	7.432	979	1.31	β-pinene	DM
	4	7.681	986	3.91	6-Methyl-5-hepten-2-one	Ketone
	5	8.732	1019	12.54	D-Limonene	Mm
	6	8.791	1032	0.50	Cineole	DLM
	7	9.218	1037	0.90	Ocimene	Cm
	8	10.506	1099	2.30	Linalool	Cm
	9	11.824	1152	0.82	Citronellal	Cm
	10	12.542	1185	2.16	Cis-Verbenone	DLM
	11	12.756	1190	0.52	α-Terpineol	MA
	12	13.984	1210	68.23	Citral	Cm
Total contents percentage of identified compounds: 96.09%						
Unidentified compounds: 3.91%						
FLEO	1	6.405	929	1.95	α-Pinene	DM
	2	6.755	952	0.92	Camphene	DM
	3	7.444	979	2.11	β-pinene	DM
	4	7.693	986	3.87	6-Methyl-5-hepten-2-one	Ketone
	5	8.744	1031	11.96	D-Limonene	Mm
	6	8.803	1032	0.70	Cineole	DLM
	7	10.518	1099	3.87	Linalool	Cm
	8	11.830	1153	0.73	Citronellal	Cm
	9	12.115	1168	0.61	Cis-Verbennol	DLM
	10	12.441	1177	0.43	Terpinen-4-ol	MA
	11	12.548	1190	0.68	α-Terpineol	MA
	12	14.774	1270	66.69	Citral	Cm
Total contents percentage of identified compounds: 94.52%						
Unidentified compounds: 5.48%						
CEO	1	7.800	1013	1.05	Hydrazinecarboxamide	HC
	2	8.738	1075	5.79	D-Limonene	Mm
	3	13.949	1142	35.36	Cis-Verbenol	DLM
	4	14.714	1276	55.29	Citral	Cm
Total contents percentage of identified compounds: 97.49%						
Unidentified compounds: 2.51%						
RO	1	7.683	988	0.71	α-Pinene	DM
	2	8.088	991	0.68	Decanoic acid	SFA
	3	9.086	1010	5.27	β-Caryophyllene	DS
	4	12.478	1189	0.51	Epoxycaryophyllene	Triterpenes
	5	16.688	1191	7.60	Palmitic acid	SFA
	6	18.355	1249	21.52	Linoleic acid	UFA
	7	18.432	1265	45.30	Oleic acid	MFA
	8	18.561	1279	1.39	Linolenic acid	PFA
	9	18.638	1286	3.80	Stearic acid	SFA
	10	20.846	1398	4.25	Cis-11-Eicosenoic acid	UFA
	11	21.219	1424	1.15	Arachidic acid	SFA
	12	24.862	1530	6.06	Erucic acid	MFA
	13	25.506	1597	0.51	Docosanoic acid	SFA
Total contents percentage of identified compounds: 98.75%						
Unidentified compounds: 1.25%						

(FREO: fresh fruits essential oil; FLEO: fresh flower essential oil; CEO: commercial *Litsea* oil; RO: rapeseed oil; PFA: Polyunsaturated fatty acid; MFA: Monounsaturated fatty acid; UFA: Unsaturated fatty acid; SFA: Saturated fatty acid; Cm: Chain monoterpene; DLM: Double loop monoterpene; HC: Hydrazine compound; DS: Dicyclic sesquiterpene; MA: Monoterpene alcohol; Mm: Monocyclic monoterpene; DM: Dicyclic monoterpene).

**Table 2:** Inhibitory rate of EOs of fresh flower and fresh fruit of *L. pungens* against two kinds of pathogenic bacteria.

Essential Oil Type		IR (%)	The Concentration of Essential Oil (mg/mL)						
	Strains		CK	CK1	CK2	2.5	5	10	20
FREO	*Bacillus anthracis*		0 ± 0.00 f	0 ± 0.00 f	85.77 ± 2.85 b	12.17 ± 1.95 e	23.10 ± 2.42 d	38.38 ± 2.00 c	100 ± 0.00 a
	*Fusarium oxysporium*		0 ± 0.00 f	0 ± 0.00 f	90.12 ± 2.45 b	5.40 ± 1.72 e	26.23 ± 2.97 d	74.74 ± 1.25 c	100 ± 0.00 a
FLEO	*Bacillus anthracis*		0 ± 0.00 e	0 ± 0.00 e	85.65 ± 4.72 a	5.62 ± 1.49 d	7.41 ± 1.18 c	13.03 ± 0.80 b	86.05 ± 2.29 a
	*Fusarium oxysporium*		0 ± 0.00 f	0 ± 0.00 f	90.12 ± 3.45 b	6.63 ± 2.01 e	19.96 ± 3.26 d	42.90 ± 1.34 c	92.45 ± 3.69 a

CK: blank control; CK1: negative control; CK2: positive control; IR: inhibition rate. Note: Values are presented as the mean ± standard error of the mean (SEM; *n* = 3). Different lowercase letters (a–f) indicate statistically significant differences (*p* < 0.05).

## Data Availability

Data is contained within the article.
