# Correction: Chen et al. Chemical Composition of *Litsea pungens* Essential Oil and Its Potential Antioxidant and Antimicrobial Activities. *Molecules* 2023, *28*, 6835

**DOI:** 10.3390/molecules31122178

**Published:** 2026-06-22

**Authors:** Tao Chen, Qingbo Kong, Xuekun Kuang, Jiasi Zhou, Haizhou Wang, Lijun Zhou, Hongyu Yang, Shiling Feng, Chunbang Ding

**Affiliations:** College of Life Science, Sichuan Agricultural University, Ya’an 625014, China; chentao293@163.com (T.C.); kqb666666@163.com (Q.K.); larrymercer11@gmail.com (X.K.); zhoujiasi1999@163.com (J.Z.); wanghaizhou4678@163.com (H.W.); zhoulijun@sicau.edu.cn (L.Z.); yhy4868135@163.com (H.Y.); fengshilin@outlook.com (S.F.)

**Error in Table**

In the footnote of the original Table 2, the data were not clearly indicated in the form of mean ± standard error of the mean (SEM) [1]. The footnote of the revised Table 2 now provides an explanation: All data are presented as mean ± SEM (*n* = 3), which is consistent with the content in Section 3.7 (Statistical Analysis) of the “Materials and Methods” section. The footnotes of Table 2 and the corresponding content in Section 3.7 have been updated in the revised version. 

**Correction to Experimental Methods**

In the published version, the end time for calculating the inhibition rate was not clearly defined. The revised Section 3.6 now clearly states: All culture plates were tested for inhibition rate 96 h after inoculation, which was the day before the blank control plate was completely covered by mycelia. 

All authors confirm that these modifications do not affect the manuscripts’ scientific conclusions. This correction has been approved by the Academic Editor, and the original published content has also been updated.
